# Enhanced wintertime greenhouse effect reinforcing Arctic amplification and initial sea-ice melting

**DOI:** 10.1038/s41598-017-08545-2

**Published:** 2017-08-16

**Authors:** Yunfeng Cao, Shunlin Liang, Xiaona Chen, Tao He, Dongdong Wang, Xiao Cheng

**Affiliations:** 1 The College of Forestry, Beijing Forestry University, 100083 Beijing, China; 2 Department of Geographical Sciences, University of Maryland, 20742 College Park, USA; 3 Department of Hydraulic Engineering, Tsinghua University, Beijing, China; 4 School of Remote Sensing and Information Engineering, Wuhan University, Wuhan, Hubei 430079 China; 5 State Key Laboratory of Remote Sensing Science, and College of Global Change and Earth System Science, Beijing Normal University, 100875 Beijing, China

## Abstract

The speeds of both Arctic surface warming and sea-ice shrinking have accelerated over recent decades. However, the causes of this unprecedented phenomenon remain unclear and are subjects of considerable debate. In this study, we report strong observational evidence, for the first time from long-term (1984–2014) spatially complete satellite records, that increased cloudiness and atmospheric water vapor in winter and spring have caused an extraordinary downward longwave radiative flux to the ice surface, which may then amplify the Arctic wintertime ice-surface warming. In addition, we also provide observed evidence that it is quite likely the enhancement of the wintertime greenhouse effect caused by water vapor and cloudiness has advanced the time of onset of ice melting in mid-May through inhibiting sea-ice refreezing in the winter and accelerating the pre-melting process in the spring, and in turn triggered the positive sea-ice albedo feedback process and accelerated the sea ice melting in the summer.

## Introduction

Despite an apparent hiatus in global warming^1–3^, the Arctic climate continues to experience unprecedented changes. Summer sea ice is retreating at an accelerated rate^4, 5^, and surface temperatures in this region are rising at a rate double that of the global average, a phenomenon known as Arctic amplification^6, 7^. Several major competing hypotheses have been proposed to explain the causes of Arctic amplification and summer sea-ice retreat. For instance, an enhanced Atlantic Meridional Overturning Circulation (AMOC) transporting extraordinary amounts of heat northward to the Arctic Ocean has been hypothesized to substantially amplify Arctic warming and accelerate summer sea-ice melting in this region^8–11^. However, evidence has instead shown significant slowing of the AMOC over the last few decades^12, 13^. Sea-ice albedo feedback caused by the continuously shrinking summer sea ice potentially increasing the absorption of shortwave radiation is also believed to play a critical role in recent Arctic amplification^14–17^. But certain model simulations have indicated that sea-ice albedo feedback was likely not the dominant factor^18, 19^ - robust warming amplification still occurs in the Arctic in the absence of albedo feedback^20, 21^. Recently, downward longwave radiation (LWD) at the surface has been suggested as an important driver of Arctic winter warming and summer sea-ice dynamics^19, 22–26^. Because LWD is affected by several highly correlated climatic factors, including atmospheric temperature, and the amounts of water vapor and cloudiness^27^, which of these factors has been the fundamental force driving Arctic amplification is still under debate. Based on model simulations, several studies have claimed that atmospheric temperature feedback (also called lapse-rate feedback at the top of the atmosphere), which is associated with wintertime temperature inversions in the Arctic boundary layer, is the dominant factor responsible for amplifying Arctic surface warming^18, 28^. The other two greenhouse effect factors, water vapor and cloudiness, have very limited (even negative) effects on Arctic amplification^17, 18^. However, other analyses, based on observations^24, 29^ and atmospheric reanalysis^23, 25^, have pointed in the opposite direction and demonstrated that the cloud- and water vapor-induced greenhouse effect is crucial to Arctic winter warming and the development of summer sea ice. In fact, LWD is much more sensitive to water vapor anomalies at high latitudes^30–33^, which suggests that the water vapor effect in the Arctic may be considerably stronger than that at lower latitudes. However the findings that support this hypothesis are either from small regions^23^ or very short spans of time (early winter^24, 25^ or late spring^23, 34^), and therefore are insufficient and problematic for fully interpreting the mechanism that drives variation in LWD at the surface and its effects on Arctic amplification and sea-ice variations. Strong and comprehensive additional evidence is therefore required to clarify the relationship between LWD at the surface and Arctic amplification.

Furthermore, because the state of sea ice at the onset of the melting season is crucial for evaluating surface energy uptake^35, 36^ which largely determines the minimum sea-ice coverage reached in the fall^37^, and is asynchronous with Arctic amplification, which occurs mainly in the wintertime^6, 15^, it is important to chronologically investigate the causal relationships between Arctic wintertime amplification and initial sea-ice melting to elucidate the physical mechanisms of recent Arctic warming.

For this study, we use for the first time, long-term (1984–2014) spatially complete satellite data to provide strong observational evidence that the enhanced greenhouse effect from increased atmospheric water vapor and cloudiness in both winter and spring may reinforce the amplification of Arctic wintertime warming, which in turn has triggered the accelerated sea-ice melting in the summer.

## Results and Analysis

**Figure 1:**
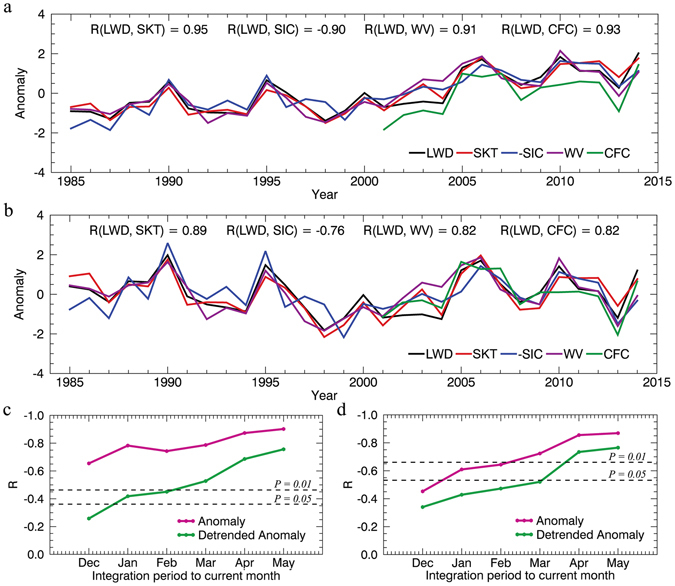
Anomalies in five spatially averaged variables for the Arctic Ocean, and the correlation coefficients between integrated surface LWD and inverted onset SIC (-SIC anomaly). Anomalies in surface LWD, SKT, CFC, and WV are averaged from December to May, and the inverted initial SIC from May 16 to June 5, (**a**) before and (**b**) after de-trending. All time series were normalized based on their corresponding standard deviations. Panels (**c**) and (**d**) show the correlation coefficients between integrated surface LWD from December to a given month and the SIC at onset of melting from May 16 to June 5 during the periods (**c**) 1985–2014 and (**d**) 2001–2014. This figure has been created with IDL8.3 (Exelis Visual Information Solutions, Boulder, Colorado).

To investigate the relationships among atmospheric longwave radiative forcing, wintertime surface warming and the late spring initial sea-ice melting in the Arctic, we calculated long-term anomalies in spatial (maximum sea-ice coverage north of 60°N) and wintertime (including both winter and spring) temporally averaged surface downward longwave radiative flux (LWD), water vapor (WV), cloud area fraction (CFC), skin temperature (SKT), and sea ice concentration (SIC) for the melting onset period^37^ between May 16 and June 5 (days of year 136 to 156). In addition, we identified the correlations between these variables from records of the last 30 years (1985–2014) (Fig. 1a and b, SIC shown here represent inverted records). The wintertime SKT of the Arctic Ocean is strongly correlated (r = 0.95, and 0.89 after de-trending, p < 0.001) with the surface LWD, and both exhibit a pronounced upward trend over the 30-year study period. Given LWD is the main source energy at the Arctic surface in boreal wintertime and a continually weakening AMOC^12, 13^ would unlikely transport extraordinary heat northward to the Arctic, the strong coupling of surface LWD and SKT imply that both inter-annual and long-term changes in SKT in the Arctic have been closely associated with surface LWD over this period. Surface LWD is related to three main parameters: temperature, clouds and water vapor^18, 28^. Therefore, the high correlation coefficients between wintertime LWD and WV over 1985–2014 (r = 0.91, and 0.82 after de-trending, p < 0.001), and between wintertime LWD and CFC over 2001–2014 (r = 0.93, and 0.82 after de-trending, p < 0.001; a high correlation was also detected for 1985–2000, Fig. S10) imply the likelihood that it is the enhanced greenhouse effect from water vapor and cloudiness that caused the increase of surface LWD, and in turn reinforced the Arctic wintertime warming over the past several decades. It is indicated that poleward moisture fluxes into the Arctic associated with large-scale circulations significantly contributed to the variation of precipitable water vapor in the Arctic^38, 39^. We also examined the relationship between the injected moisture across 70°N and the total precipitable water vapor in the Arctic (Fig. S11), the strong correlation between the two variables demonstrate that the variation of the Arctic water vapor is primarily controlled by the transported convergence of moisture from the lower latitude. The poleward transported moisture would bring not only water vapor, but also latent energy into the Arctic^23, 38^.

To investigate the influence of wintertime surface LWD on the state of sea ice at the onset of melting, we directly calculated the correlation between wintertime LWD and the melting onset SIC in late spring. As shown in Fig. 1a and b, the total wintertime surface LWD is strongly negatively correlated with the subsequent onset SIC (r = −0.90, and −0.76 after de-trending) in late spring. Arctic sea ice usually begins to refreeze in early October and attains its maximum coverage in early March^40, 41^ before entering an ice pre-melting state until the onset of melting in mid-May^40^. The gradual increase in correlation coefficients between the integrated surface LWD and onset SIC (Fig. 1c,d, two correlation maps are also generated and shown in Fig. S12) demonstrates that the accumulated wintertime surface LWD potentially influences both the ice refreezing and pre-melting processes through reducing ice thickness^25^, and the perturbing of ice thickness would in turn affects the SIC during the period of melting onset (especially over these coastal regions with thin ice)^42^. After sea-ice melting begins, shortwave albedo feedback becomes more important on driving variations in ice coverage over the subsequent months^35, 36^. The conclusions of this study, based on long-term observational evidence, are partially consistent with previous studies that have reported isolated cases of atmospheric conditions influencing ice melting in either the late spring^23, 29, 34^ or winter^24, 25^. However, our results indicate that the downward longwave radiation from early winter to late spring is continually affecting both the inter-annual and long-term changes of the Arctic sea ice state.

To quantify the contributions of greenhouse effects associated with water vapor and cloudiness to the long-term trend of LWD, and in turn Arctic wintertime warming and initial sea-ice melting, we have calculated the statistical linear trends of all variables for both the winter and spring seasons and estimated the surface downward longwave radiative forcing caused by changes in water vapor and cloudiness. As shown in Table 1, the significantly positive trends of cloudiness and water vapor in the Arctic wintertime (from December to May) can generate a total of 2.99 W m^−2^ per decade (0.61 W m^−2^ per decade from cloudiness and 2.38 W m^−2^ per decade from water vapor) in additional longwave radiative forcing, and the sum of the two totally contributed 81% of the linear trend of LWD (3.70 W m^−2^ per decade). This finding indicates that the increase of wintertime surface LWD in the Arctic is mainly resulted from the enhanced greenhouse effect by increased amounts of water vapor and cloudiness. Therefore, temperature feedback mechanisms, particularly thermal inversions during the winter, are unlikely to be the dominant factor driving the amplification of Arctic wintertime warming^18, 28, 43^. The trend during winter (4.13 W m^−2^, 56%) is close to that of spring (3.28 W m^−2^, 44%), which indicates that the atmospheric greenhouse effects in the winter and spring seasons are both important to the sea-ice initial melting in late spring.

**Table 1:** Statistical linear trends of surface downward longwave radiation (LWD), skin temperature (SKT), precipitable water vapor (WV), WV radiative forcing (F_wv_), cloud fraction (CFC), cloud radiative forcing (CRF) and onset SIC from 1985 to 2014.

Time Period	Variables	Trend (per decade)	Confidence level (%)
Winter (Dec – Feb)	LWD	4.13 W m^−2^	99.97
	SKT	1.35 K	>99.99
	WV	0.20 mm	99.99
	F_wv_	3.25 W m^−2^	99.99
	CFC	2.77%	66.97
	CRF	0.93 W m^−2^	41.30
Spring(Mar – May)	LWD	3.28 W m^−2^	99.98
	SKT	0.90 K	>99.99
	WV	0.16 mm	99.84
	F_wv_	1.89 W m^−2^	99.84
	CFC	2.03 (4.82)%	99.94(97.69)
	CRF	0.30 W m^−2^	19.75
Wintertime (Dec – May)	LWD	3.70 W m^−2^	>99.99
	SKT	1.12 K	>99.99
	WV	0.18 mm	>99.99
	F_wv_	2.38 W m^−2^	>99.99
	CFC	3.79%	96.98
	CRF	0.61 W m^−2^	48.34
Melting onset (May 16 – June 05)	SIC	−2.69%	>99.99

Values marked with underline are the linear trends from 2001 to 2014. Confidence level is the complement of the significance (here, p-value based on F-test) of a linear trend, calculated as “(1 - significance) × 100%”.

**Figure 2:**
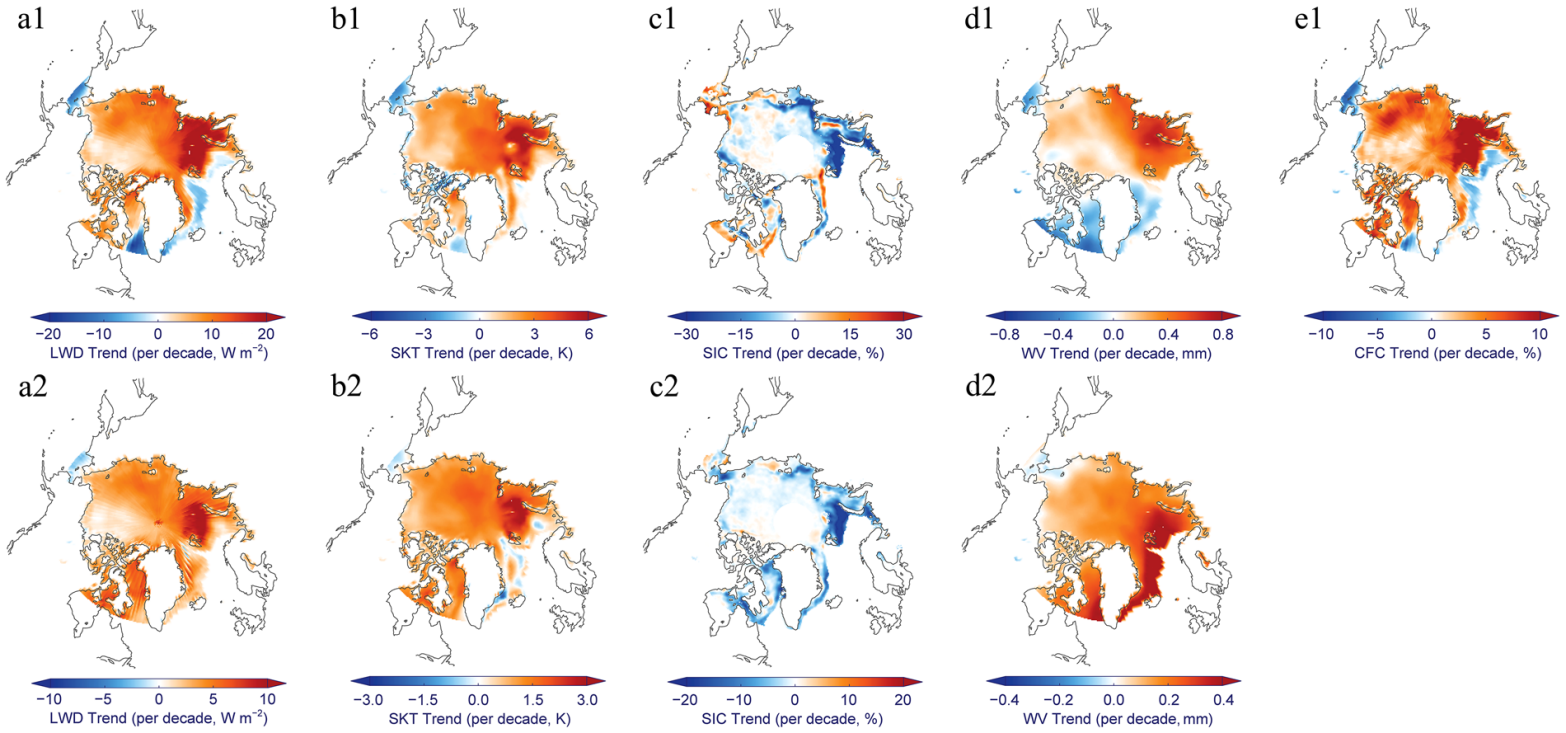
Observed trends for all five variables. Linear trends in (**a1,a2**) surface LWD, (**b1, b2**) SKT, (**c1,c2**) onset SIC, (**d1,d2**) WV, and (**e1**) CFC from 2001 to 2014 (upper panels) and from 1985 to 2014 (lower panels). This figure has been created with IDL8.3 (Exelis Visual Information Solutions, Boulder, Colorado) and the open source Coyote Library developed by David Fanning (https://github.com/idl-coyote/coyote).

The strong linkages between all five variables can be further verified through the coupling of spatially distributed linear trends from 2001–2014 (Fig. 2, upper panels) and 1985–2014 (Fig. 2, bottom panels). The varying spatial patterns of all four variables from 1985 to 2014 are very similar (Fig. 2, bottom panels), and further confirm the likelihood that the enhanced wintertime surface LWD has substantially warmed the Arctic ice surface over the last 30 years and resulted in dramatic sea-ice retreat during seasons of melting onset. In a number of hotspots, such as the Barents-Kara Sea, northern Canada and the Greenland Sea, large increases in surface LWD by up to 5–10 W m^−2^ per decade over the last 30 years resulted from the significant increases in water vapor and cloudiness have warmed the ice surface by 1.5–3 K per decade, and highly likely then induced a decline in onset SIC by 10–20% per decade. Over the last 14 years (Fig. 2, upper panels), warming in the Arctic, and especially in the Barents-Kara Sea, has become even more pronounced; the trends of LWD and SKT have nearly doubled, and the decline in onset SIC has also accelerated. In contrast, warming in Baffin Bay was likely interrupted, and the decline in onset SIC there slowed down, with even a slight increase in the southern part of this region.

We also explored the relationships among all of these variables based on inter-annual variations in the de-trended time series. With a threshold of ±0.5 standard deviations for the de-trended melting onset SIC time series, we detected eight low-onset ice years (LOIYs) and nine high-onset ice years (HOIYs). We then calculated the averaged anomalies of all variables for LOIYs and HOIYs and estimated the surface downward longwave radiative forcing from changes in water vapor and cloudiness (Table 2). In LOIYs, the above-average greenhouse effect from positive cloudiness and water vapor anomalies in the Arctic wintertime season (December to May) can together generate approximately 3.04 W m^−2^ (0.81 W m^−2^ from cloudiness and 2.23 W m^−2^ from water vapor) extra downward longwave radiative forcing, which is almost equal to the surface LWD anomaly of 3.22 W m^−2^. These findings indicate that the greenhouse effect caused by water vapor and cloudiness also plays a dominant role in the inter-annual variation of Arctic wintertime LWD, and then skin temperature and initial sea-ice melting. The contribution of the winter season (2.94 W m^−2^, 46%) is similar to that of the spring season (3.50 W m^−2^, 54%), which indicates that the atmospheric greenhouse effects in both the winter and spring are important to initial sea-ice melting in the Arctic.

**Table 2:** Statistical anomalies in surface downward longwave radiation (LWD), skin temperature (SKT), precipitable water vapor (WV), WV radiative forcing (F_wv_), cloud fraction (CFC), cloud radiative forcing (CRF) and onset SIC for LOIYs.

Time Period	Variables	LOIYs Anomaly	Confidence level (%)
Winter (Dec – Feb)	LWD	2.94 W m^−2^	98.04
	SKT	0.80 K	97.68
	WV	0.13 mm	97.31
	F_wv_	2.18 W m^−2^	97.31
	CFC	1.25%	93.82
	CRF	0.50 W m ^−2^	92.90
Spring (Mar – May)	LWD	3.50 W m^−2^	98.49
	SKT	0.84 K	98.74
	WV	0.20 mm	99.12
	F_wv_	2.28 W m^−2^	99.12
	CFC	1.50%	95.64
	CRF	1.13 W m ^−2^	98.03
Wintertime (Dec – May)	LWD	3.22 W m^−2^	99.74
	SKT	0.82 K	98.87
	WV	0.17 mm	99.52
	F_wv_	2.23 W m^−2^	99.52
	CFC	1.22%	97.04
	CRF	0.81 W m ^−2^	97.91
Melting onset (May 16 – June 05)	SIC	−2.28%	99.97

The confidence level for each variable is based on a Monte Carlo approach with 10,000 iterations, similar to a previous study by^23^. Values marked with underline are the statistical anomalies from 2001 to 2014.

**Figure 3:**
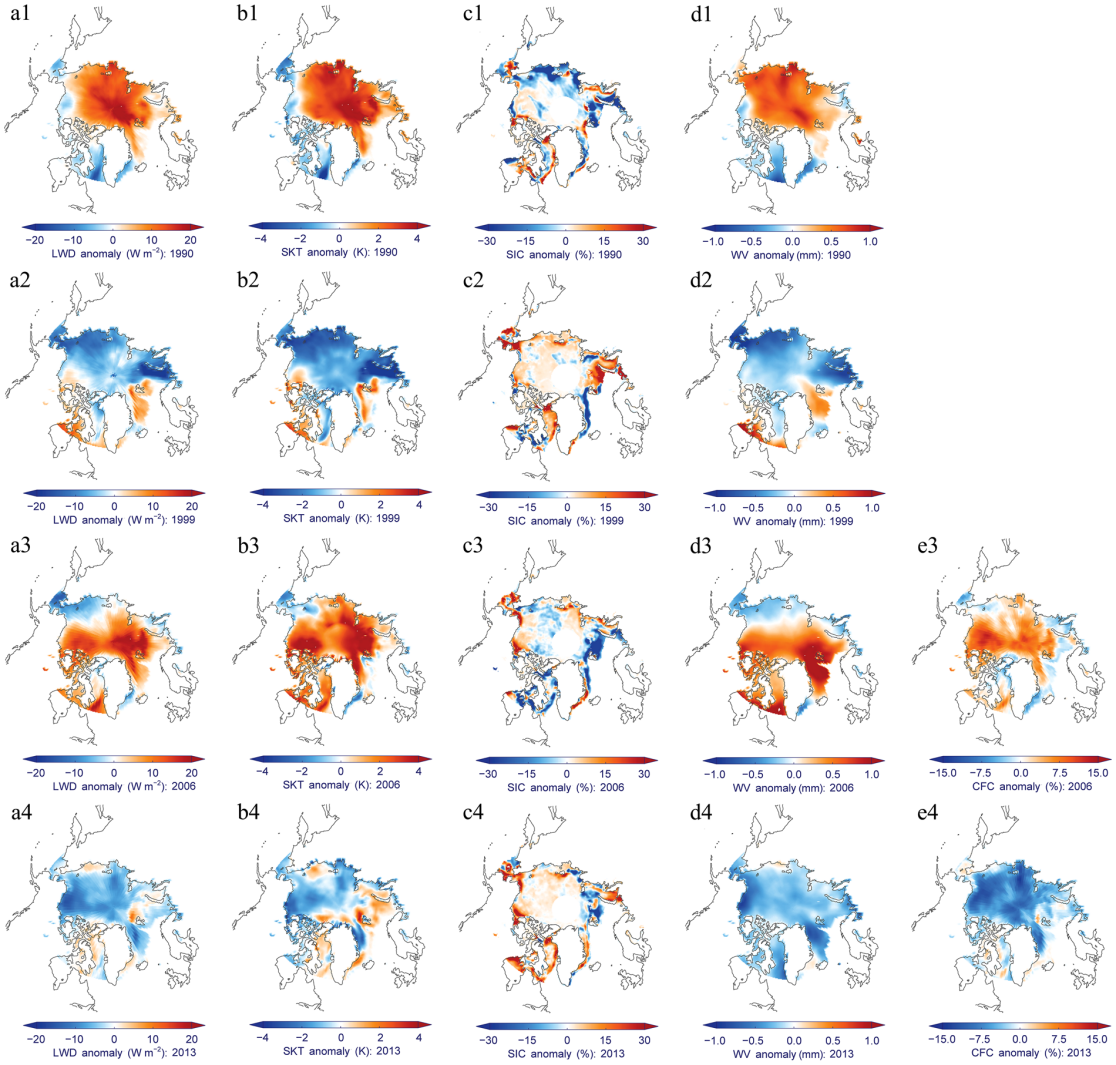
Spatial patterns in de-trended anomalies of all five variables for four representative years. Anomalies in surface (**a1–a4**) LWD, (**b1–d4**) SKT, (**c1–c4**) initial SIC, (**d1–d4**) WV and (**e3–e4**) CFC in 1990, 1999, 2006 and 2013, respectively. This figure has been created with IDL8.3 (Exelis Visual Information Solutions, Boulder, Colorado) and the open source Coyote Library developed by David Fanning (https://github.com/idl-coyote/coyote).

The results for four representative years, comprising two low-onset ice years, 1990 and 2006, and two high-onset ice years, 1999 and 2013, were selected to explore the coupling characteristics of the spatial patterns of the de-trended anomalies for all five variables (Fig. 3). The high spatial consistency of anomalies in surface LWD, SKT, WV, CFC and onset SIC is reflected in both the overall spatial pattern and many local details. For instance, in the low-onset ice years (1990 and 2006), the above-average precipitable water vapor and cloudiness caused extraordinarily high wintertime surface LWD, which resulted in higher SKT across most areas of the Arctic Ocean, and then these anomalies quite likely led to lower initial sea-ice coverage in these regions. In the high-onset ice years (1999 and 2013), below-average levels of precipitable water vapor and cloudiness produced weak atmospheric downward longwave radiative flux and brought a colder Arctic wintertime climate. These anomalies might result in more extensive sea-ice refreezing before March, and less melting in early spring, which then lead to greater sea-ice coverage at the onset of the ice-melting season. The highly similar spatial patterns of all five variables for both warmer and colder years support the hypothesis that changes in the energy exchange between ice and the overlying atmosphere highly likely controls the thermodynamic mechanism that affect sea ice in the wintertime^24^, and therefore the state of sea ice at the onset of the melting season. Since only the thin ice over coastal regions begin to melt in late spring^40^, and the sea-ice state is also influenced by surface wind^10^, ocean currents^9^, ice albedo feedback^35^ and other factors^18^, there are still some differences in the spatial patterns of both long-term trend and de-trended anomalies between onset SIC and other four variables.

## Discussion

The strong linkages between precipitable WV, CFC, surface LWD and SKT in both the temporal and spatial distributions of anomalies demonstrates that the recent Arctic wintertime amplification, which occurs mainly in the winter and spring, is strongly related to an enhanced greenhouse effect from increased water vapor and cloudiness. Quantification of surface downward longwave radiative forcing produced by changes in the amounts of water vapor and cloudiness verified that the greenhouse effect associated with these two variables controls both inter-annual variation and long-term trend of the wintertime surface downward longwave radiation in the Arctic, and then the skin temperature. The strong evidence we describe contrasts with the results of model simulations described in previous studies that indicated relatively limited contributions of water vapor and cloudiness to Arctic surface warming compared to the feedback effects associated with temperature inversion^18, 28^. In model simulations, contributions from cloudiness and water vapor are only considered from a view of feedback mechanism, while a growing body of studies suggest that most of the increased water vapor over the Arctic is transported from lower latitudes^23, 38, 44, 45^.

Furthermore, our findings suggest that the enhanced greenhouse effect from increased cloudiness and water vapor in the Arctic has highly likely inhibited the process of ice refreezing in winter, and has accelerated the pre-melting process in early spring, and then resulted in an advance in the melting onset of sea ice in the late spring and triggered the shortwave albedo feedback process in the latter melting season. The influence of the Arctic atmosphere on sea ice is therefore an ongoing process from early winter to late spring. Previous studies that have accounted only for the effects of surface LWD on ice dynamic in either winter^24, 25^ or late spring^23, 29, 34^ have been insufficient for understanding the chronological linkage between atmospheric conditions and sea-ice variation. Our data fusion algorithm has enabled us to obtain a long-term, high quality surface downward longwave radiation dataset to solve this problem. And these findings in this study elucidate the underlying major physical mechanisms of recent Arctic wintertime warming and its forcing on initial ice melting in the Arctic.

## Data and Methods

### Radiative flux datasets

The Clouds and the Earth’s Radiant Energy System (CERES) Synoptic Radiative Fluxes and Clouds (SYN) product, version 3 A, has been evaluated on a global scale, and is considered to be the best source of observed surface downward longwave radiative fluxes currently available^27^. This data product has been recommended by the CERES Science Team as the best resource available for estimation of surface fluxes^46^. However, this dataset includes data only for years after 2000. There are additional datasets, either from reanalysis or from satellite observations (Table S1), that include earlier periods to as early as the 1980s. To take advantage of both the highly accurate CERES-SYN surface radiative flux data as well as the longer-period coverage of multi-source datasets, a data fusion algorithm was designed, validated and applied for this study with the objective of generating a more accurate, continuous, consistent and long-term dataset of downward longwave radiative flux at the surface. The non-negative least squares (NNLS) model^47^, which is a well-developed approach that has been widely used in image fusion studies^48, 49^, was applied in the data fusion algorithm. The most significant characteristic of the NNLS model is its use of non-negativity constraints that leads to parts-based representation because it allow only additive, not subtractive, combination of individual components. A detailed description of the data fusion methodology is included in the supplementary materials. Based on the validation results (Fig. S2–S9), the multi-source data fusion algorithm performs very well compared to the CERES SYN radiative flux data, and the fused dataset yields substantial improvement across all months, as indicated by much larger R squares and lower root-mean-square errors (RMSEs) and biases than those of any other input dataset. The NNLS data fusion algorithm was therefore applied to generate a fused dataset for the years 1984–2000. By combining the fused 1984–2000 dataset with the CERES SYN data for 2001–2014, we have obtained a continuous, consistent surface downward longwave radiation dataset for 1984–2014.

### Cloud fraction and water vapor datasets

Cloud area fraction data for 2000–2014 from the CERES SYN 1.0° product derived using the CERES-MODIS (Moderate Resolution Imaging Spectroradiometer) cloud retrieval algorithm, and 1.0° cloud fraction data for 1984–2007 from the Global Energy and Water Cycle Experiment Surface Radiation Budget (GEWEX SRB^50^) derived from the International Satellite Cloud Climatology Project (ISCCP) DX product^51^ are used in this study. The monthly 0.67° × 0.50° precipitable water vapor dataset from Modern Era Retrospective-Analysis for Research and Applications (MERRA) used in this study was produced by NASA’s Global Modeling and Assimilation Office Goddard Earth Observing System Data Assimilation System, version 5 (GEOS-5) using a three-dimensional variational data assimilation (3D-Var) framework with an emphasis on the hydrologic cycle^52^. An incremental analysis update (IAU) procedure and a variety of recent satellite observations, such as NASA’s Earth Observing System, SSM/I radiances, TIROS Operational Vertical Sounder (TOVS) radiances, Atmospheric Infrared Sounder (AIRS) radiances and scatterometer wind retrievals, were incorporated into the assimilation system to improve estimates of the global energy and water budgets^22, 52^.

### Sea-ice datasets

The fourth version of the Equal-Area Scalable Earth Grid (EASE-Grid) 2.0 weekly snow cover and sea ice extent and the daily sea-ice concentration product, derived from the Scanning Multichannel Microwave Radiometer (SMMR) onboard Nimbus-7, and the Special Sensor Microwave Imager and the Special Sensor Microwave Imager Sounder (SSM/I-SSMIS) onboard the Defense Meteorological Satellite Program (DMSP), provided by the National Snow and Ice Data Center (NSIDC) are both used in this study^53, 54^. The weekly sea-ice extent data were applied to identify the maximum area of sea-ice coverage from 1984 to 2014 to define the study area.

### Ice surface temperature dataset

We also used the ERA-Interim monthly temperature data product^55^, which has been successfully utilized in many previous studies^6, 15, 56, 57^. The ERA-Interim skin temperature dataset used here has similar properties to the surface 2-meter air temperature dataset^58^. The ERA-Interim surface temperature dataset was extensively validated with *in-situ* measurements at high latitudes, and was found to outperform all other available reanalysis products^59, 60^. The quality of this dataset is comparable even to observations from satellites over the Arctic land surface^58^.

### Spatially averaged statistics

To calculate spatially averaged statistics for the Arctic Ocean, two separate phases were executed. The first phase was mask generation; the mask of maximum sea-ice coverage for the area north of 60°N between 1984 and 2014 (180°W−180°E for the Arctic Ocean) were derived using the Equal Area Scalable Earth Grid (EASE-Grid) 2.0 Weekly Sea Ice Extent data product. The mask was used to define the study area. In the second phase, all grid datasets (radiative flux, sea surface temperature, precipitable water vapor, and cloud fraction) were first re-projected onto an equal-area projection, and then the maximum sea-ice coverage mask was applied to calculate spatially averaged values.

### Calculation of surface LWD forcing by precipitable water and cloudiness

To quantify the contributions of greenhouse effects from precipitable water vapor and cloudiness, we calculated the surface LWD forcing generated by both precipitable water vapor and cloudiness separately. For the estimation of water vapor radiative forcing, we apply a formula for this relationship based on *in-situ* radiation flux measurements and water vapor datasets^33^:

1 $$\bigtriangleup {F}_{WV}=173.1\times [{(WV+\bigtriangleup WV)}^{0.22}-W{V}^{0.22}]$$

Here, *WV* and ${\rm{\Delta }}{WV}$ are the climatology and anomaly of precipitable water vapor, ${\rm{\Delta }}{F}_{{wv}}$ is the anomaly of surface LWD caused by the change in the amount of water vapor. The cloud radiative forcing (CRF) was calculated as the all-sky surface LWD minus the clear-sky surface LWD based on the CERES SYN radiative flux datasets.

The confidence level of a statistical anomaly in Table 2 is tested using a Monte Carlo approach with 10,000 iterations, as done in a previous study^23^. For each iteration, we first randomly generate 10,000 LOIY/HOIY artificial composites, and then compare to the original LOIY/HOIY anomaly composite. The confidence level of an original composite is calculated as one minus the percentage of the absolute values of the artificial composites larger than the original anomaly composite. This method is thought to be more robust than a Student t-test approach that requires data follow a normal distribution.

## Electronic supplementary material

Supplementary Information
