# Supplementary material for: Enhanced wintertime greenhouse effect reinforcing Arctic amplification and initial sea-ice melting
Source: Sci Rep. 2017 Aug 16;7:8462. doi: 10.1038/s41598-017-08545-2 (PMC5559487; doi:10.1038/s41598-017-08545-2)
Supplement: Supplementary file 1 — Supplementary Information [file 41598_2017_8545_MOESM1_ESM.pdf]

Supplementary Information

Enhanced wintertime greenhouse effect reinforcing Arctic amplification and initial sea-ice melting

Yunfeng Cao[1] Shunlin Liang[2,*] Xiaona Chen[3] Tao He[4,2] Dongdong Wang[2] Xiao Cheng[5]

[1]The College of Forestry, Beijing Forestry University, 100083, Beijing, China

[2]Department of Geographical Sciences, University of Maryland, 20742 College Park, USA

[3]Department of Hydraulic Engineering, Tsinghua University, Beijing, China

[4]School of Remote Sensing and Information Engineering, Wuhan University, Wuhan, Hubei 430079, China

[5]State Key Laboratory of Remote Sensing Science, and College of Global Change and Earth System Science, Beijing Normal University, 100875 Beijing, China

[*]Corresponding author: Department of Geographical Sciences, University of Maryland, 20742 College Park, USA (sliang@umd.edu)

1. Multi-source data fusion methodology

The data fusion algorithm aims to integrate the multi-source products for generating a more accurate, continuous, consistent and long-term surface radiative flux dataset. To take advantage of the high quality of CERES-SYN surface radiative flux and the long temporal coverage of other seven datasets (Table S1), we built and validated the data fusion model using the dataset from 2000 to 2007, and then applied the model to the datasets from 1984 to 2000, the framework was illustrated in Fig. S1.

Table S1 The surface radiative flux products used in data fusion process.

Data Set	Resolution	Temporal span	References
GEWEX_SRB	1.0° × 1.0°	1983.07 – 2007.12	[*Stackhouse et al.*, 2011]
ISCCP	2.5° × 2.5°	1983.07 – 2009.12	[*Rossow and Robert*, 1999]
CLARA_A1	0.25° × 0.25°	1982.01 – 2009.12	[*Karlsson et al.*, 2013]
ERA-Interim	1.0° × 1.0°	1979.01 – 2013.12	[*Dee et al.*, 2011]
MERRA	0.67° × 0.50°	1979.07 – 2013.12	[*Rienecker et al.*, 2011]
CFSR	0.5° × 0.5°	1979.07 – 2013.12	[*Saha et al.*, 2010]
JRA-55	0.56° × 0.56°	1979.07 – 2013.12	[*Kobayashi et al.*, 2015]

There are several phases for the data fusion work consisting of the data preprocessing, model generation, model validation, model application and data combination.

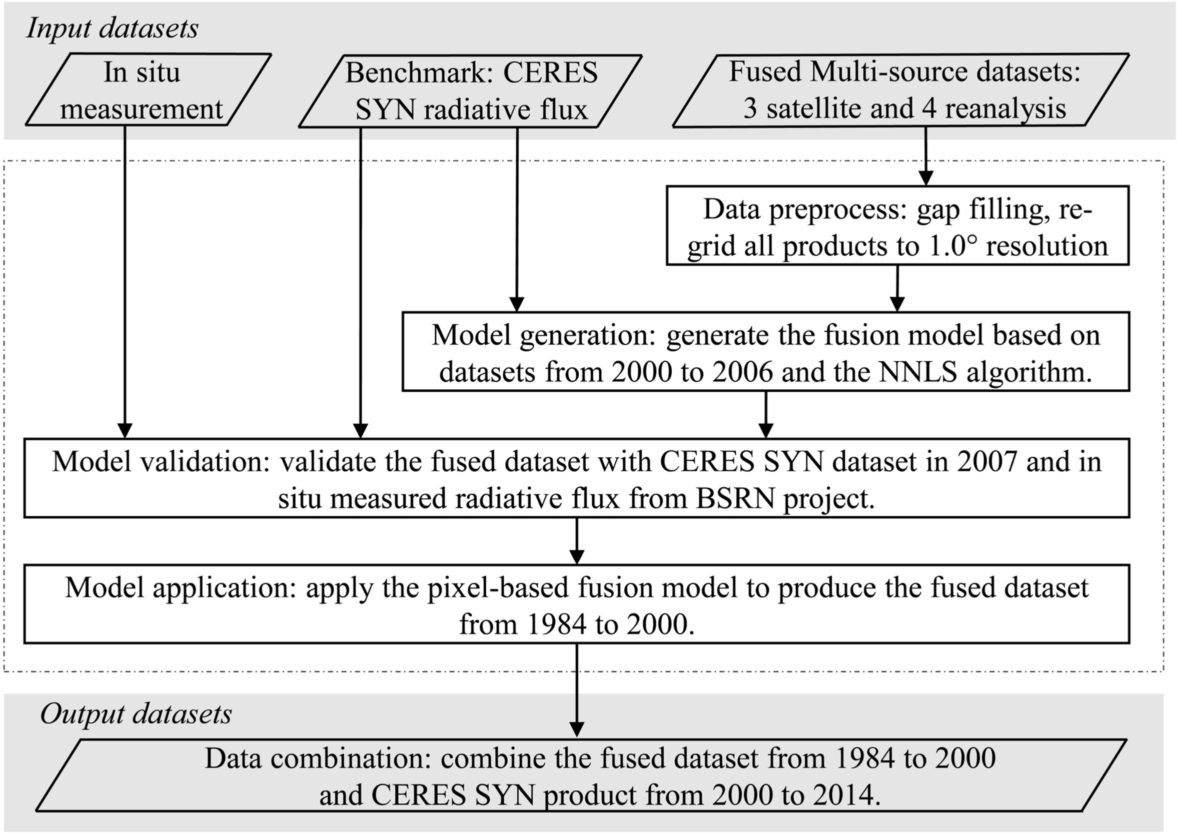

Fig. S1. Flowchart of the data fusion process.

In the data preprocessing phase, we created a quality control for every product to fill the gaps with a temporal filter process; all products were then re-gridded to 1.0° resolution.

The non-negative least square (NNLS) [*Bro and Sijmen*, 1997] regression approach, which is a very mature and widely used approach in image fusion studies [*Lee and Seung*, 1999; *Patrik*, 2004], was used in building the data fusion model with datasets from 2000 to 2006. We chose the non-negative multiple linear model because the non-negativity constraint of NNLS only allow an additive, not subtractive, combination of all components. Given radiative flux are always positive, a key assumption here is that the benchmark radiative flux can be expressed as a non-negative linear combination of other 7 products at each grid, one product may present very little of the real flux variation, but should not have a negative contribution. To take advantage of the

spatial similarity characteristics of the radiative flux (in a small window, pixels show similar values and inter-annual variations), a 3×3-pixel local window was used to train the model, and each local model is used equally on all nine pixels inside the window. With a moving model generation process, nine groups of model coefficients were generated for every grid. The final model was built on averaging the nine group model coefficients. So as to avoid the influence from seasonal cycle on the expression of the inter-annual variation of radiative flux, the models were generated monthly.

2. Validation of the multi-source fused datasets with CERES SYN product

The fused surface downward longwave radiative flux was validated using the CERES SYN dataset in 2007, which was not involved in the model generation process. The validated results were shown in Figs. S2-7.

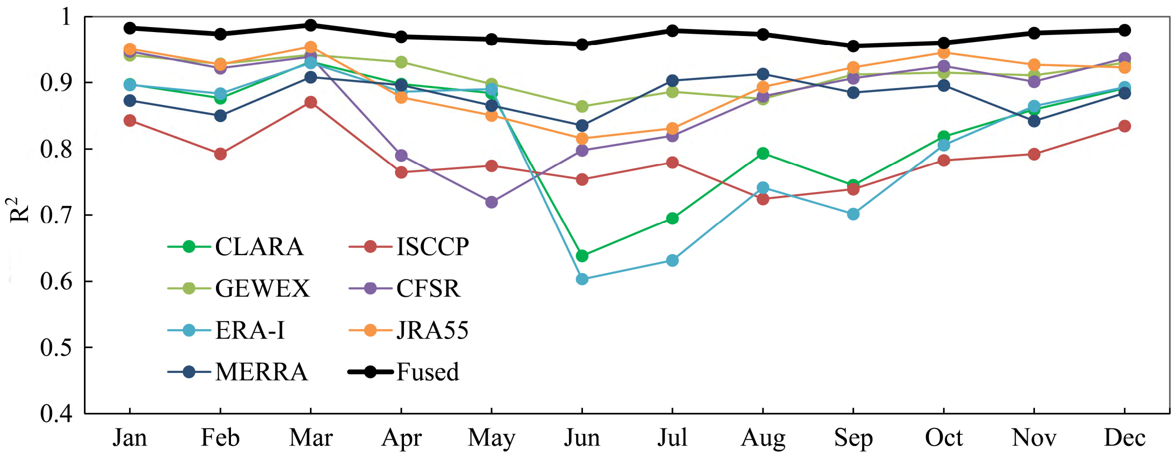

Fig. S2. R^2 for each validated dataset relative to CERES-SYN product. It indicates that the NNLS fusion model can significantly improve the accuracy and consistency of fused dataset. The fused dataset show higher R squares in all months than any other single product.

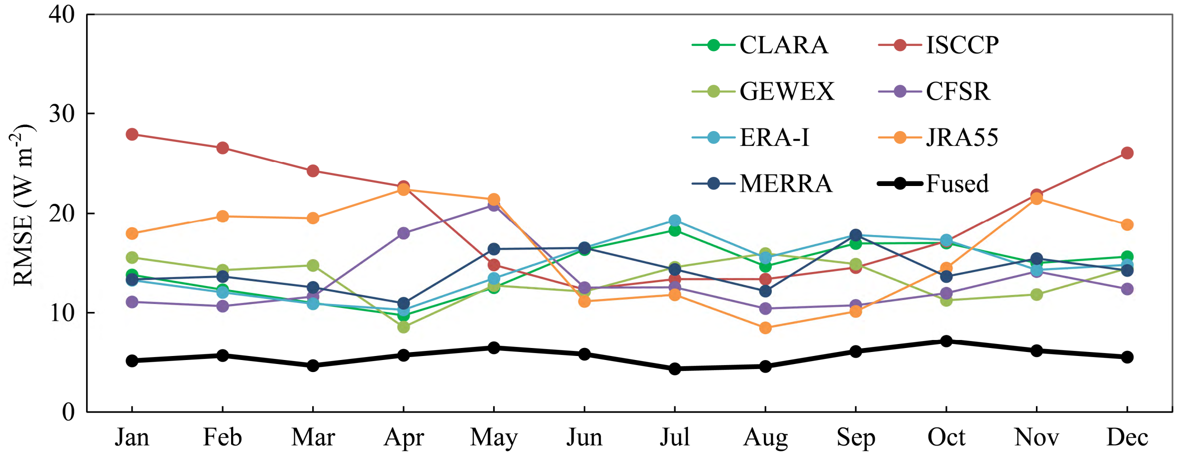

Fig. S3. RMSE for each validated dataset relative to CERES SYN product. The fused dataset have lower root-mean-square-error (RMSE) than any other single product, show higher consistency with CERES SYN product.

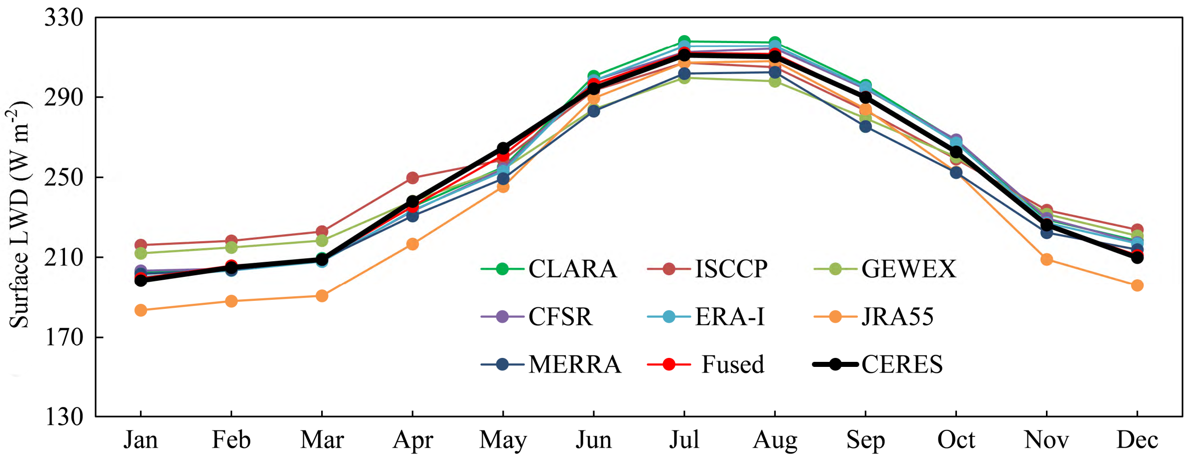

Fig. S4. Seasonal cycle of all datasets in the Arctic. The fused datasets with NNLS model agree well with CERES SYN product. Especially in the wintertime months from December to May focused in this study, the fused datasets perform even better than any other input product.

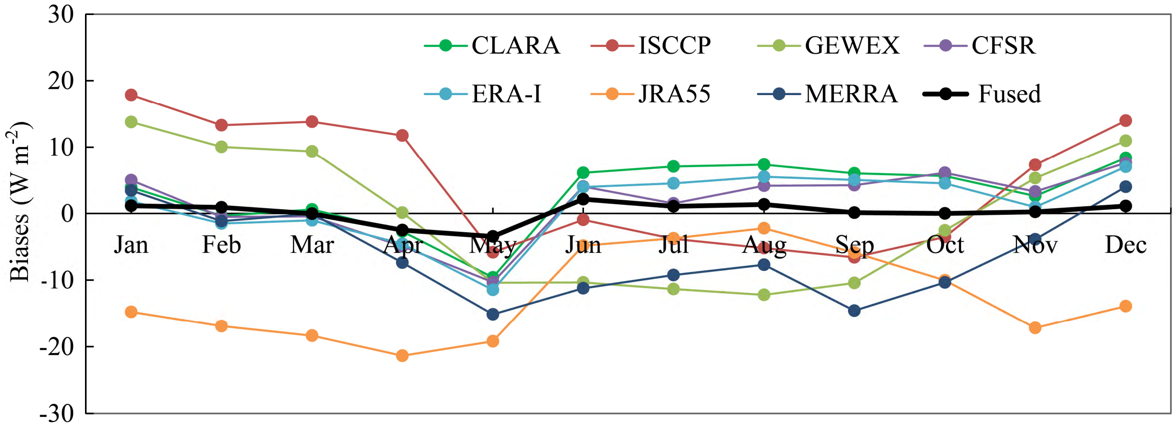

Fig. S5. Monthly biases of all datasets in the Arctic relative to CERES SYN product. The fused datasets with the NNLS model consistent well with CERES SYN product. The biases in all months were reduced significantly and even larger in these wintertime months from December to May.

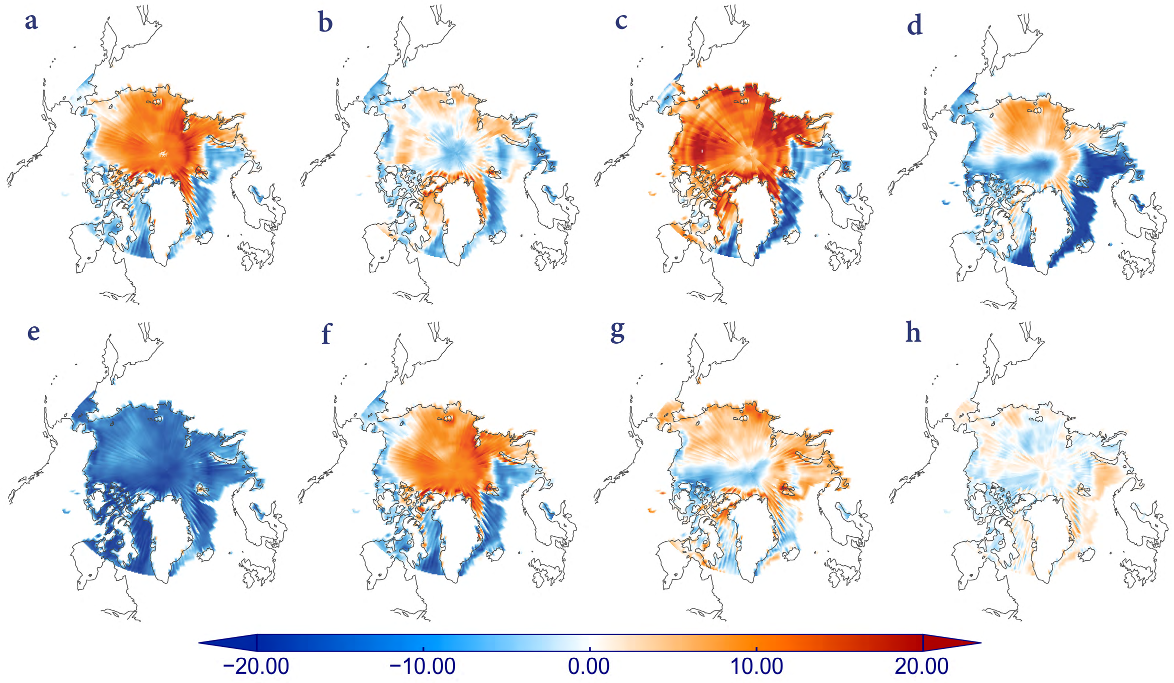

Fig. S6. Spatial pattern of annual mean surface LWD differences between all other datasets and CERES-SYN product in the Arctic: a, CLARA-A1; b, GEWEX; c, ISCCP; d, MERRA; e, JRA55; f, ERA-Interim; g, CFSR; h, Fused NNLS. The fused dataset show lower difference with CERES SYN radiative flux product. This figure has been created with IDL8.3 (Exelis Visual Information Solutions, Boulder,

Colorado) and the open source Coyote Library developed by David Fanning (https://github.com/idl-coyote/coyote).

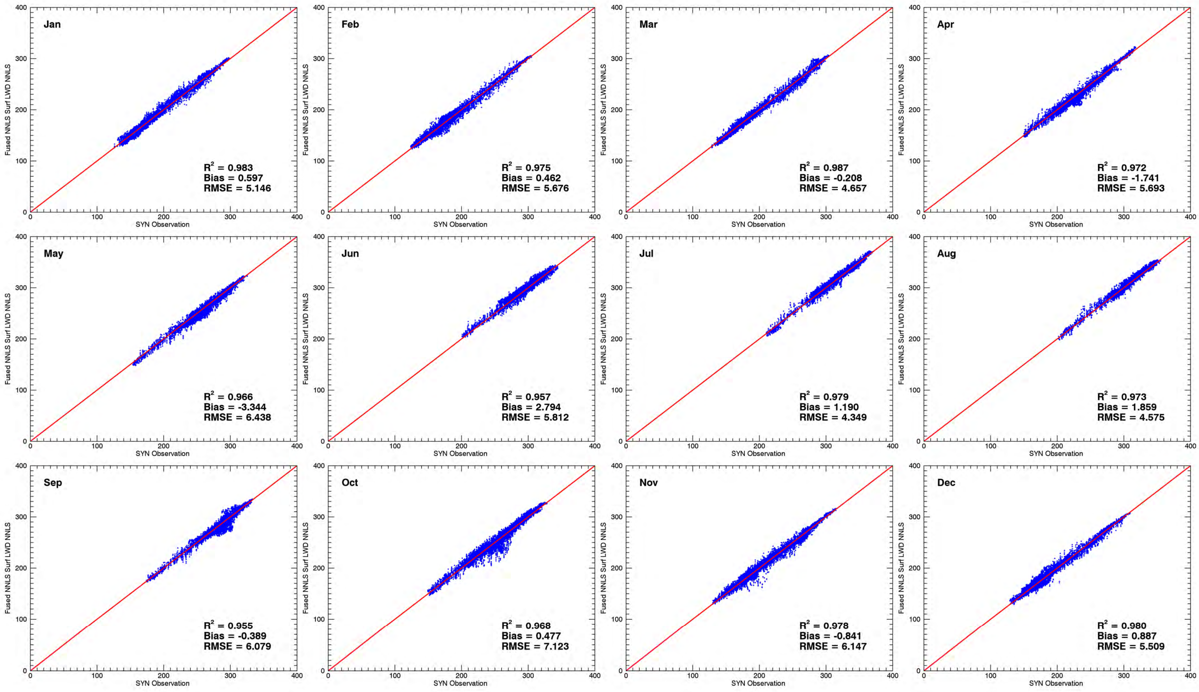

Fig. S7. Scatter plot of fused dataset and CERES SYN product in Arctic region in all twelve months. The results indicate that NNLS data fusion method performs well in all months, and even better in the wintertime months from December to May. This figure has been created with IDL8.3 (Exelis Visual Information Solutions, Boulder, Colorado).

3. Validation of the multi-source fused datasets with in situ measurements

To validate the fused dataset, the BSRN (Baseline Surface Radiation Network) ground observed dataset, which is regarded as providing the most accurate observations of surface downward longwave radiative flux [*Wang and Dickinson*, 2013] was used in this study. There are two BSRN sites (Barrow, 156.611°W, 71.323°N; Ny-Ålesund, 11.93°E, 78.925°N) in the Arctic have more than 20 years of observed data. These data allow us to evaluate both the accuracy of fused dataset and its consistency with CERES SYN product. The validation results shown in Fig. S8 and Fig. S9 demonstrate that the fused dataset not only have high absolute accuracy, but the comparable validated results of R squares, RMSEs and biases

between fused dataset and CERES SYN product show it also have similar quality to CERES SYN product.

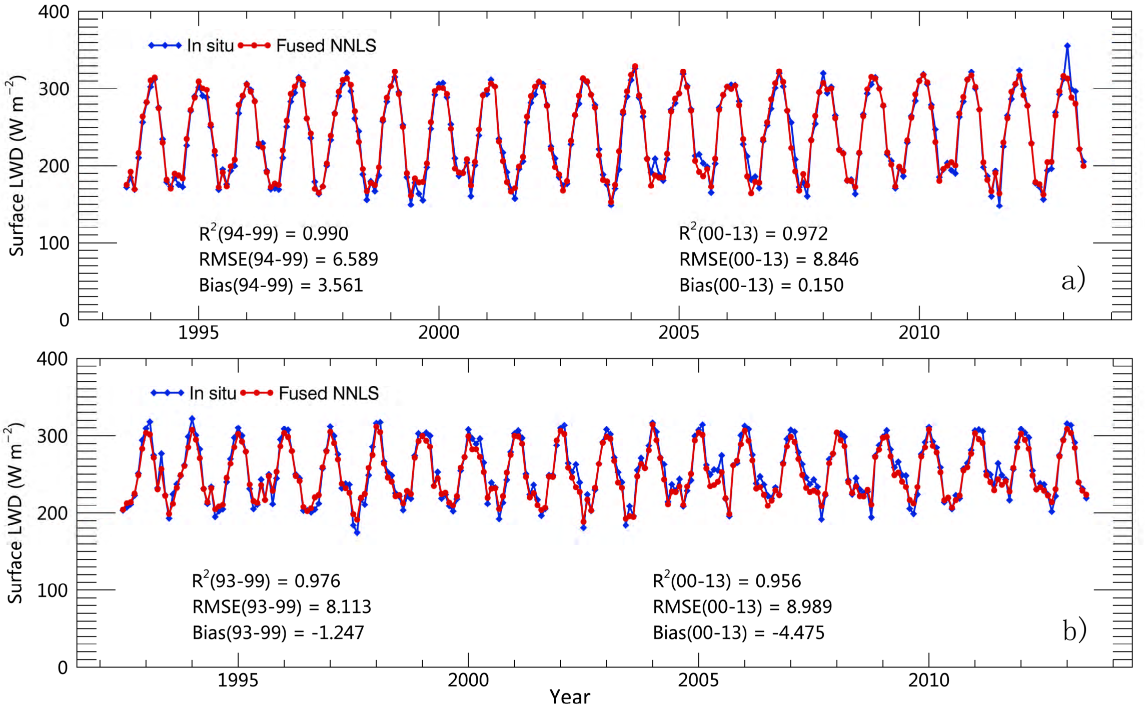

Fig. S8. Time series of fused and in situ measured surface downward longwave radiation at Barrow (a) and Ny-Ålesund (b) stations. This figure has been created with IDL8.3 (Exelis Visual Information Solutions, Boulder, Colorado).

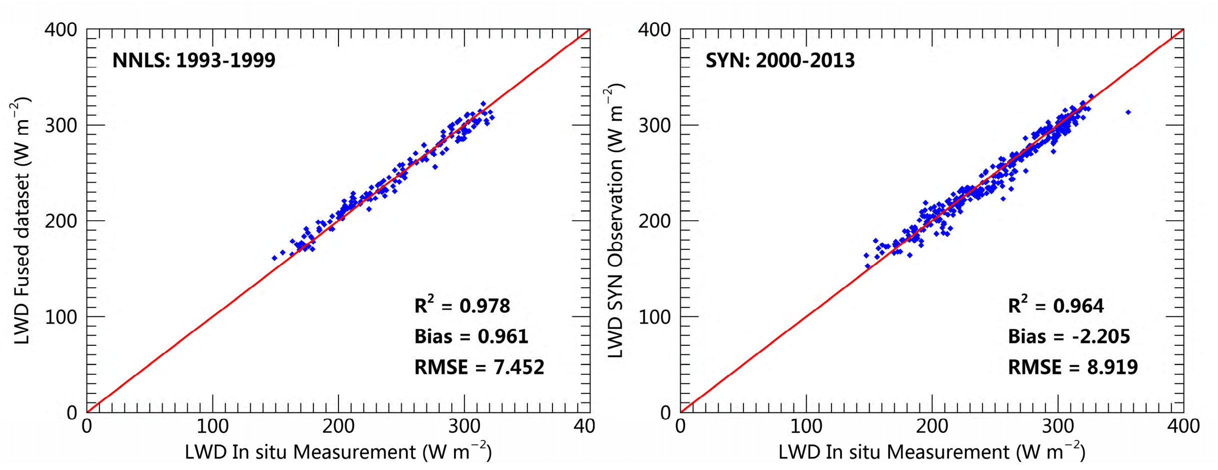

Fig S9. Scatter plots of the fused surface downward longwave radiation and all BSRN ground observations in the Arctic. This figure has been created with IDL8.3 (Exelis Visual Information Solutions, Boulder, Colorado).

4. Anomalies of surface LWD, cloud fraction averaged from March to May

Because of the large gap in GEWEX cloud fraction product in the three winter months from December to February. Instead of calculating the average value for radiative flux, cloud fraction and water vapor from December to May as in Fig. 1 of the main text, here we calculated the average of radiative flux and cloud fraction only in the three spring months. The high correlation coefficients between surface LWD and GEWEX cloud fraction during 1984 to 2000 ($r = 0.84$, and 0.80 after de-trend, $p < 0.001$), and between surface LWD and CERES SYN cloud fraction during 2000 to 2014 ($r = 0.89$, and 0.78 after de-trend, $p < 0.001$) confirm the high influence of cloudiness on surface LWD.

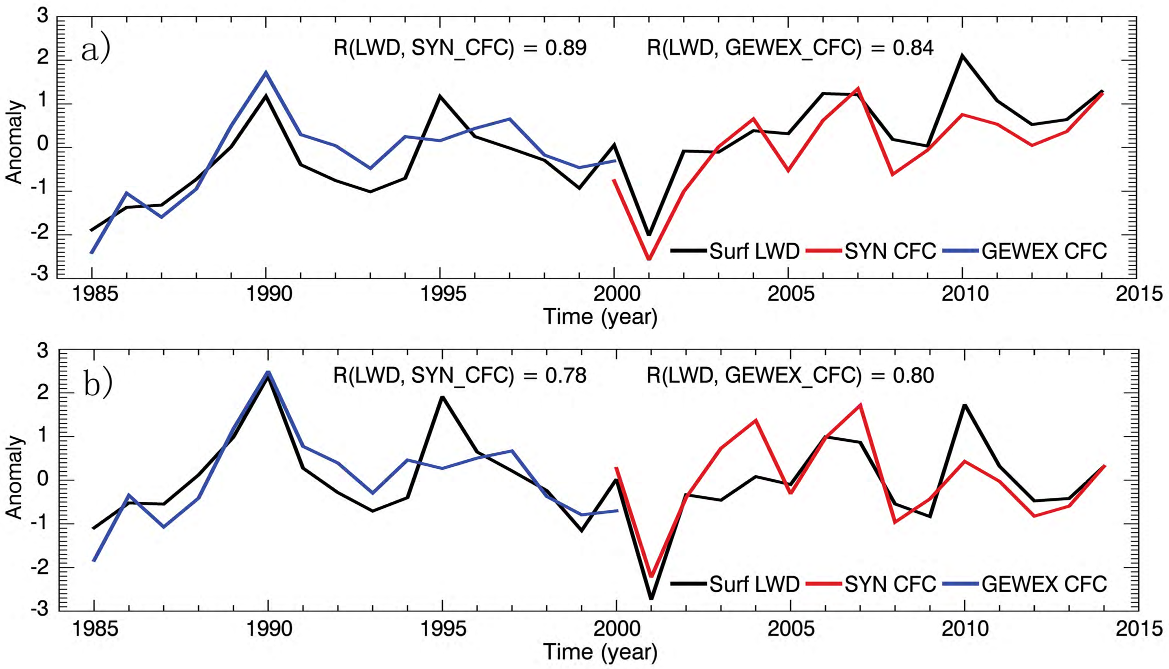

Fig. S10. Anomaly of Arctic surface downward longwave radiation and cloud fraction from GEWEX (blue, 1985 to 2000) and from CERES SYN (red, 2000 to 2014) averaged over March to May before (a) and after (b) de-trend. All time-series are normalized by the corresponding standard deviation. The high correlation coefficients between cloud fraction and surface downward longwave radiative flux before (a) and after (b) de-trend indicate that cloudiness have significant influences on both the inter-annual and long-term trend of surface downward longwave radiative flux in the last thirty years. This figure has been created with IDL8.3 (Exelis Visual Information Solutions, Boulder, Colorado).

5. Anomalies of injected moisture across 70°N and total precipitable water vapor

Based on the method proposed by previous studies [Woods and Caballero, 2016; Woods et al., 2013], the moisture injection (MI) into Arctic can be calculated as follow,

$$\mathrm{MI} = \frac{1}{g}\int_0^{p_s} vqH(v)dp \qquad \text{(S1)}$$

Here, v is the northward velocity of wind, q is the specific humidity, H() is the Heaviside function. With MERRA product, we examined the relationship between poleward transport of water vapor across 70°N and the total precipitable water vapor in the Arctic during boreal winter and spring (December to May), as shown in Fig. S11. The strong correlation coefficients between poleward moisture and water vapor in the Arctic before (r = 0.71) and after (r=0.76) de-trend indicate that the variability of the total precipitable water vapor in the Arctic is mainly controlled by poleward transport moisture from lower latitudes. The poleward transport moisture would bring both water vapor and latent energy to enhance the surface downward longwave radiation and warm the Arctic ice surface in boreal wintertime [Graversen and Burtu, 2016].

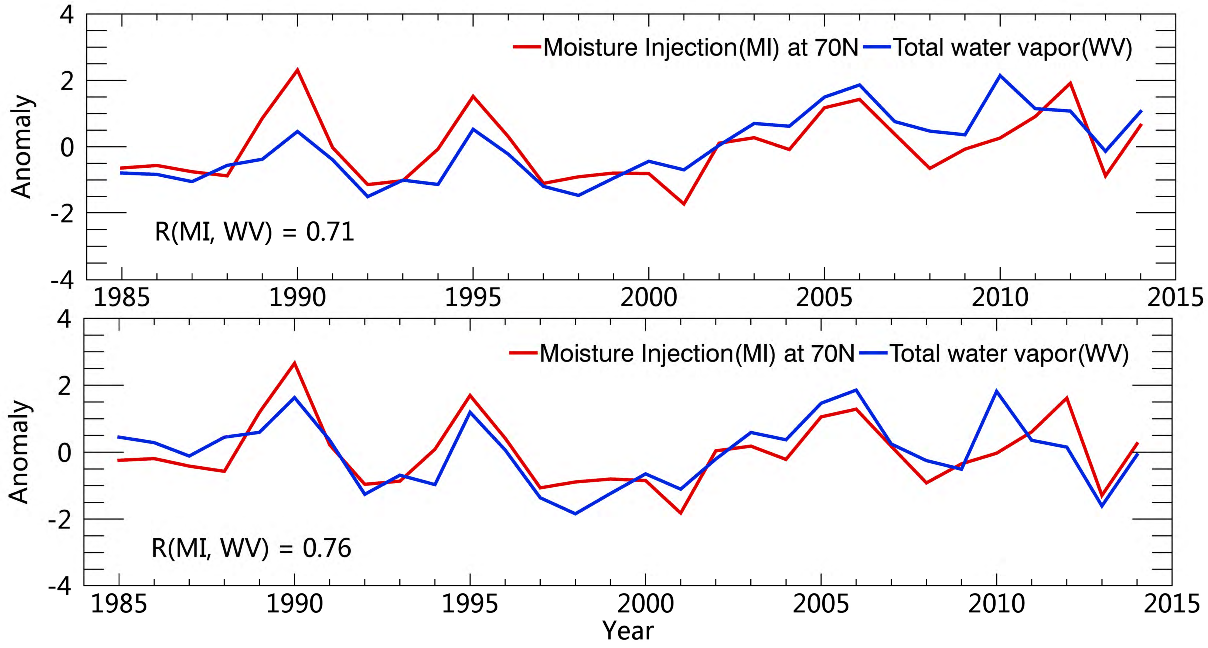

Fig. S11. Anomaly of moisture injection (MI) across 70°N and total precipitable water vapor in the Arctic averaged over December to May before (upper panel) and after (bottom panel) de-trend. All time-series are normalized by the corresponding standard deviation. This figure has been created with IDL8.3 (Exelis Visual Information Solutions, Boulder, Colorado).

6. Correlation maps between the integrated surface LWD and onset SIC

The correlation maps between integrated surface LWD and onset SIC in following figure indicates that there are still some differences in the spatial patterns between LWD and onset SIC. This is mainly because that only thin ice located at the coastal regions begin to melt in late spring[*Markus et al.*, 2009], and some other mechanisms such as surface wind[*Zhang*, 2015], ocean currents[*Spielhagen et al.*, 2011], ice albedo feedback[*Cao et al.*, 2015] also influence the Arctic sea-ice state. The onset SIC over these regions covered by perennial sea ice, although the wintertime ice thickness might be perturbed, may have little change in late spring.

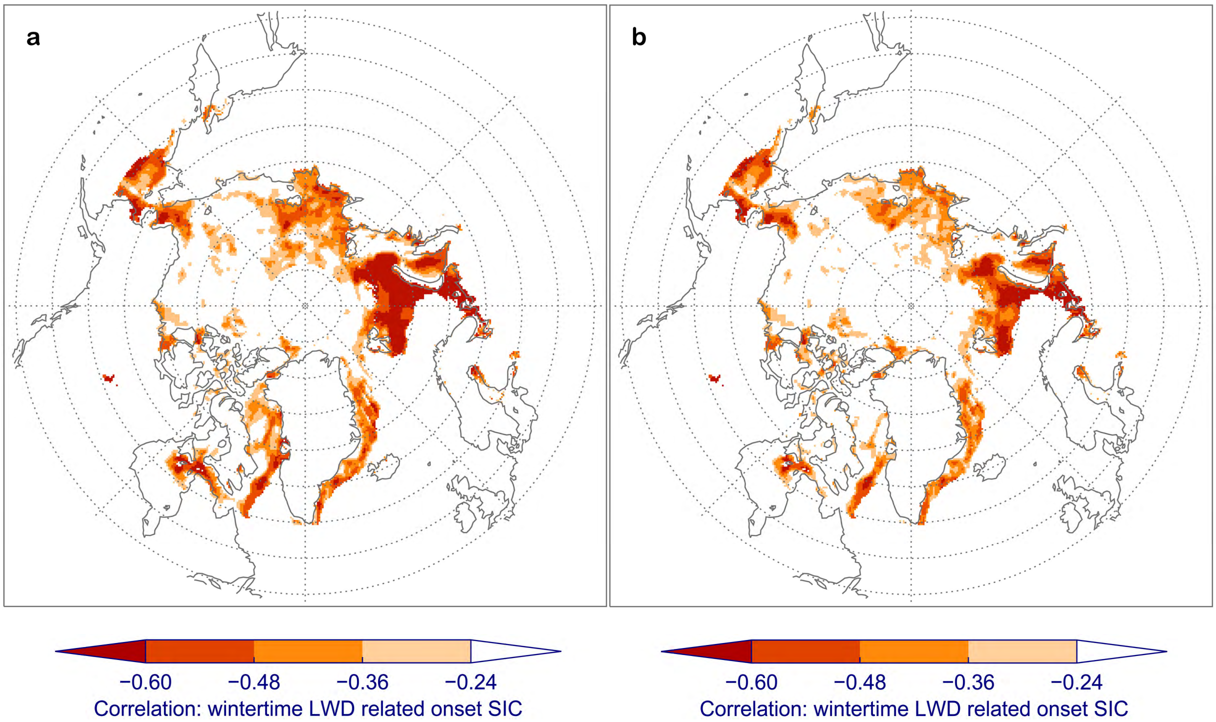

Fig. S12. Correlation maps between the integrated surface LWD from December to May and the averaged SIC from May 16 and June 5 (days of year 136 to 156) before (a) and after de-trending (b). These values lower than -0.36 are significant at 0.05 level. This figure has been created with IDL8.3 (Exelis Visual Information Solutions, Boulder, Colorado).

7. Statistical anomalies of these five variables in HOIYs.

Table S2 | Statistical anomalies of surface downward longwave radiation (LWD), skin temperature (SKT), precipitable water vapor (WV), WV radiative forcing (F_{wv}), cloud fraction (CFC), cloud radiative forcing (CRF) and onset sea ice concentration for HOIYs. Values marked with underline are the statistical anomalies from 2001 to 2014.

Time Period	Variables	HIYs Anomaly	Confidence level (%)
Winter (Dec – Feb)	LWD	-1.33 W m^{-2}	93.64
	SKT	-0.48 K	93.92
	WV	-0.09 Kg m^{-2}	94.73
	F_{wv}	-1.54 W m^{-2}	94.73
	CFC	-1.85%	96.68
	CRF	-0.20 W m^{-2}	89.51
Spring (Mar – May)	LWD	-2.69 W m^{-2}	97.12
	SKT	-0.72 K	98.23
	WV	-0.17 Kg m^{-2}	99.20

	F_{wv}	-2.04 W m^{-2}	99.20
	CFC	-1.61%	95.04
	CRF	-1.73 W m^{-2}	99.62
Pre-melting season (Dec – May)	LWD	-2.01 W m^{-2}	98.14
	SKT	-0.60 K	98.49
	WV	-0.13 Kg m^{-2}	98.49
	F_{wv}	-1.83 W m^{-2}	98.51
	CFC	-2.31%	99.63
	CRF	-0.97 W m^{-2}	98.80
Melting onset (May 16 – June 05)	SIC	1.68%	99.85

References

Bro, R., and D. J. Sijmen (1997), A fast non-negativity-constrained least squares algorithm, *J. Chemom.* , *11*(5), 393-401, doi:10.1002/(SICI)1099-128X(199709/10)11:5<393::AID-CEM483>3.0.CO;2-L.

Cao, Y., S. Liang, X. Chen, and T. He (2015), Assessment of sea-ice albedo radiative forcing and feedback over the Northern Hemisphere from 1982 to 2009 using satellite and reanalysis data, *J. Clim.*, *28*(3), 1248-1259, doi:10.1175/jcli-d-14-00389.1.

Dee, D. P., et al. (2011), The ERA-Interim reanalysis: configuration and performance of the data assimilation system, *Q. J. ROY. METEOR. SOC.*, *137*(656), 553-597, doi:10.1002/qj.828.

Graversen, R. G., and M. Burtu (2016), Arctic amplification enhanced by latent energy transport of atmospheric planetary waves, *Q. J. ROY. METEOR. SOC.*, *142*(698), 2046-2054, doi:10.1002/qj.2802.

Karlsson, K. G., et al. (2013), CLARA-A1: the CM SAF cloud, albedo and radiation dataset from 28 yr of global AVHRR data, *Atmos. Chem. Phys.* , *13*(1), 935-982, doi:10.5194/acpd-13-935-2013.

Kobayashi, S., et al. (2015), The JRA-55 Reanalysis: General Specifications and Basic Characteristics, *Journal of the Meteorological Society of Japan. Ser. II*, *93*(1), 5-48, doi:10.2151/jmsj.2015-001.

Lee, D. D., and S. H. Seung (1999), Learning the parts of objects by non-negative matrix factorization, *Nature*, *401*, 788-791, doi:10.1038/44565.

Markus, T., J. C. Stroeve, and J. Miller (2009), Recent changes in Arctic sea ice melt onset, freezeup, and melt season length, *J. Geophys. Res.* , *114*(C12), C12024, doi:10.1029/2009jc005436.

Patrik, O. H. (2004), Non-negative Matrix Factorization with Sparseness Constraints, *Journal of Machine Learning Research*, *5*, 1457-1469.

Pithan, F., and T. Mauritsen (2014), Arctic amplification dominated by temperature feedbacks in contemporary cliamte models, *Nat. Geosci.* , *7*(3), 181-184, doi:10.1038/ngeo2071.

Rienecker, M. M., et al. (2011), MERRA: NASA's Modern-Era Retrospective Analysis for Research and Applications, *J. Clim.*, *24*(14), 3624-3648, doi:10.1175/jcli-d-11-00015.1.

Rossow, B. W., and S. A. Robert (1999), Advances in Understanding Clouds from ISCCP, *Bull. Am. Meteorol. Soc.* , *80*(11), 2261-2287, doi:10.1175/1520-0477(1999)080<2261:AIUCFI>2.0.CO;2.

Saha, S., et al. (2010), The NCEP Climate Forecast System Reanalysis, *Bull. Am. Meteorol. Soc.* , *91*(8), 1015-1057, doi:10.1175/2010bams3001.1.

Spielhagen, R. F., K. Werner, S. A. Sorensen, K. Zamelczyk, E. Kandiano, G. Budeus, K. Husum, T. M. Marchitto, and M. Hald (2011), Enhanced modern heat transfer to the Arctic by warm Atlantic Water, *Science*, *331*(6016), 450-453, doi:10.1126/science.1197397.

Stackhouse, J. P. W., S. K. Gupta, S. J. Cox, T. Zhang, J. C. Mikovitz, and L. M. Hinkelman (2011), The NASA/GEWEX surface radiation budget release 3.0: 24.5-year dataset, *GEWEX News*, *21*(1), 10-12.
Wang, K., and R. E. Dickinson (2013), Global atmospheric downward longwave radiation at the surface from ground-based observations, satellite retrievals, and reanalyses, *Rev. Geophys.* , *51*(2), 150-185, doi:10.1002/rog.20009.
Woods, C., and R. Caballero (2016), The Role of Moist Intrusions in Winter Arctic Warming and Sea Ice Decline, *J. Clim.*, *29*(12), 4473-4485, doi:10.1175/jcli-d-15-0773.1.
Woods, C., R. Caballero, and G. Svensson (2013), Large-scale circulation associated with moisture intrusions into the Arctic during winter, *Geophys. Res. Lett.* , *40*(17), 4717-4721, doi:10.1002/grl.50912.
Zhang, R. (2015), Mechanisms for low-frequency variability of summer Arctic sea ice extent, *Proc. Nat. Acad. Sci. U.S.A.* , *112*(15), 4570-4575, doi:10.1073/pnas.1422296112.
